# Analysis of clinical characteristics in proximal and distal reflux monitoring among patients with gastroesophageal reflux disease

**DOI:** 10.1515/med-2023-0791

**Published:** 2023-10-11

**Authors:** Ping Wang, Jie Yu, Bing-Lin Heng, Yan Chen, Hong Guo, Ying-Jian Zhang

**Affiliations:** School of Basic Medicine and Forensic Medicine, Henan University of Science and Technology, Luoyang 471003, Henan, China; Department of Gastroenterology, The First Affiliated Hospital of Henan University of Science and Technology, Luoyang 471003, Henan, China; Department of Gastroenterology, The First Affiliated Hospital of Henan University of Science and Technology, No. 24 of Jinghua Road, Luolong District, Luoyang 471003, Henan, China

**Keywords:** distal reflux, gastroesophageal reflux disease, proximal regurgitation, 24-h cavity impedance joint pH monitoring technique

## Abstract

The purpose of this study was to examine the characteristics of proximal and distal gastroesophageal reflux in patients with gastroesophageal reflux disorder and analyze their clinical symptoms. A total of 67 patients with typical esophageal symptoms were selected for this study. All participants completed the reflux disease questionnaire and a questionnaire survey of extraesophageal symptoms. Diagnosis was made using a 24-h impedance-pH detection and proton pump inhibitor. The results showed that the proximal reflux group had a higher number of acid reflux episodes compared to the distal reflux group (*P* < 0.05). Similarly, the proximal reflux group also had a higher number of gas reflux episodes compared to the distal reflux group (*P* < 0.05). On the other hand, the distal reflux group had a higher number of mixed reflux episodes compared to the proximal reflux group (*P* < 0.05). These differences were statistically significant. This study revealed that acid reflux and gas reflux were more predominant in the proximal reflux group, while mixed reflux was more predominant in the distal reflux group.

## 1 Introduction

Gastroesophageal reflux disease (GERD) is characterized by the inadequate functioning of the lower esophageal sphincter (LES), resulting from various local or systemic causes. Here gastric and duodenal contents flow back into the esophagus resulting in a series of symptoms such as heartburn. As per the endoscopic findings, GERD is generally classified into three types, namely, non-erosive reflux disease, reflux esophagitis (RE), and Barrett’s esophagus [1]. GERD has become one of the most common gastrointestinal diseases in China, and is gaining greater attention. Typical symptoms of GERD include heartburn, acid reflux, and chest pain. Atypical symptoms encompass abdominal discomfort, chronic cough, a sensation of a foreign body in the throat, and asthma, among others [2,3].

Epidemiological data show that the incidence of GERD in western countries is higher than the Asia-Pacific region. Estimates suggest that the prevalence of GERD in European and American countries ranges from 10 to 15%, while in Asia, it is reported to be around 5% [4]. The most recent report from China estimates the prevalence to be 3.8% [5].

The pathogenesis of GERD is multifaceted and typically encompasses the following aspects [6]: (1) The pressure of LES is reduced, and the anti-reflux barrier function is weakened. Using esophageal pressure measurement, Hou et al. found that the pressure of LES was decreased in 59% of the 27 patients with GERD in their study. (2) Esophageal peristalsis dysfunction leads to decreased esophageal clearance ability; (3) It involves impairment of the esophageal mucosal barrier function; (4) The diaphragm participates in the anti-reflux barrier; (5) Angle of His; (6) Mental and psychological factors. At the same time, diabetes is also closely related to the incidence of GERD [7]. With improvements in living standards, changes in dietary patterns, and increased daily life stress and work pressures, there has been a global increase in GERD cases in recent years [8].

The 24-h esophageal pH monitoring is a crucial diagnostic method for GERD. This monitoring involves continuous pH monitoring of the esophagus over 24 h under normal physiological conditions. It helps determine if the patient has pathological acid reflux, assess the severity of reflux, examine the pattern of acid reflux throughout the day and night, establish the relationship between acid reflux and symptoms, and assess the patient’s response to treatment. It was previously considered the “gold standard” for diagnosing GERD [9]. Currently, GERD diagnosis mainly relies on the scoring system developed by Johnson and DeMeester [10].

Recent studies have highlighted the significance of weak acid and weak base reflux in GERD. Xu et al. [11] found that weak alkaline reflux also plays an important role in the pathogenesis of GERD. However, the traditional 24-h ambulatory esophageal pH monitoring technology can only measure changes in H+ concentration and cannot detect non-acid reflux (weak acid and weak base reflux), let alone the composition of reflux. Therefore, with the increasing challenge of diagnosing GERD, 24-h esophageal multichannel intraluminal impedance monitoring combined with pH-metry (24 h MII-pH) technology has been widely used in the diagnosis of GERD. 24-h MII-pH monitoring is considered to be the most sensitive method for monitoring reflux events, which can not only help identify whether the reflux is acid reflux, weak acid reflux, or weak base reflux but can also distinguish whether the reflux is liquid, gas, or mixed reflux. This method enables the detection of various types of esophageal reflux characteristics, including fluid, gas, acid, and non-acid characteristics [12].

Currently, due to its limited popularity, high equipment cost, and poor patient tolerance, 24-h esophageal impedance pH monitoring is not commonly available for most GERD patients.

Proximal and distal reflux: These refer to the reflux of impedance channel 15 and 5 cm above LES, respectively. 24-h MII-pH monitoring not only can help monitor the reflux 5 cm above LES, but can also monitor the reflux 15 cm away from the proximal end of LES. However, there is currently no consensus on the reference standard for proximal esophageal reflux.

The findings of these studies indicate that proximal reflux episodes could potentially contribute to respiratory symptoms [13]. There is evidence that the proximal esophagus seems to be more sensitive to the distal esophagus than to heartburn and chest pain (typical symptoms) caused by mechanical dilation, electrical stimulation, [14] and acid reflux. A recent study [15] observed that the afferent nerves in the proximal mucosa are shallower compared to those in the distal region. This anatomical difference may explain the heightened sensitivity to symptoms induced by proximal esophageal reflux.

## 2 Materials and methods

### 2.1 Study participants

In this study, we recruited patients with typical esophageal symptoms, including acid reflux, heartburn, chronic cough, and sensation of a foreign body in the throat, from the Department of Gastroenterology, the First Affiliated Hospital of Henan University of Science and Technology, Luoyang, Henan, China between November 2018 and October 2019. We included patients aged 18–80 years, regardless of gender, and with chief complaints that included extraesophageal symptoms.

Respondents completed questionnaires on reflux disease (the Reflux Disease Questionnaire or the RDQ) and extraesophageal symptoms [16] under the guidance of physicians, and we calculated the RDQ score. GERD was indicated when the total score was ≥12, and 24-h MII-pH monitoring was done. Based on the symptom scores and 24-h MII-pH monitoring results, we divided the patients into the proximal reflux group and the distal reflux group, with 67 cases in total, consisting of 40 males and 27 females.

Inclusion criteria: (1) Patients who obtained a total score of ≥12 on the RDQ; (2) Patients capable of cooperating with 24-h MII-pH monitoring; (3) Patients within the age range of 18–80 years; (4) Patients who had not taken acid inhibitors, antacids, or prokinetic drugs in the 2 weeks prior to the examination; (5) Among patients reporting a sensation of a foreign body in the throat, only those whose laryngoscopy indicated no significant injury to the throat mucosa were included; (6) Patients with chronic cough whose chest digital radiography results were normal; (7) Patients without a history of neck, nasopharynx, or gastrointestinal surgery.

Exclusion criteria: (1) Pregnant and lactating women; (2) Patients who had participated in a clinical trial within the past 3 months or were currently enrolled in a trial; (3) Chronic cough caused by other factors such as respiratory tract infection or chronic bronchitis; (4) Patients whose sensation of a foreign body in the throat was attributed to other causes, such as chronic pharyngitis or chronic tonsillitis; (5) Patients who had received anti-acid and prokinetic drugs within 2 weeks prior to the examination; (6) Patients with a previous history of neck, nasopharynx, or gastroesophageal surgery.

### 2.2 General information

We collected various general details from the participants, including age, gender, place of residence, height, weight, smoking history, drinking history, previous surgery history, as well as information on proton pump inhibitor’s (PPI) dosage and duration.

### 2.3 RDQ and extraesophageal symptom questionnaire

The RDQ was used to evaluate the severity and frequency of acid reflux, heartburn, retrosternal pain, and reflux. The severity of symptoms was evaluated using a 5-point rating scale: 1 for asymptomatic, 2 for very mild symptoms that did not significantly impact daily life, 3 for moderately noticeable symptoms occasionally affecting daily activities, 4 for symptoms ranging between moderate and severe, and 5 for severe symptoms significantly impacting daily life and activities.

The frequency of symptoms was rated using a 5-point scale as follows: 0 if the symptom never occurred; 1 if the symptoms lasted less than 1 day in the last 1 week; 2 if the frequency of the symptom in the last 1 week was for 1 day; 3 if the symptom in the last 1 week lasted for 2–3 days; 4 if the symptom occurred on 4–5 days within the last 1 week; 5 if the symptom occurred on 6 days or was felt every day within the last 1 week. The overall RDQ score was calculated by summing the scores for symptom severity and frequency. A score of ≥12 points indicated a diagnosis of GERD (Tables 1–3).

**Table 1:** Symptom frequency of reflux disease questionnaire

Symptom	Never	<1 day in 1 week	1 day in 1 week	2 or 3 days in 1 week	4 or 5 days in 1 week	6 or 7 days in 1 week
Heartburn	0	1	2	3	4	5
Acid reflux	0	1	2	3	4	5
Food reflux	0	1	2	3	4	5
Chest pain	0	1	2	3	4	5

**Table 2:** Symptom severity of reflux disease questionnaire

Symptom	Asymptomatic	Slight	Obvious	Heavier	Severe
Heartburn	1	2	3	4	5
Acid reflux	1	2	3	4	5
Food reflux	1	2	3	4	5
Chest pain	1	2	3	4	5

**Table 3:** Atypical symptoms and extraesophageal symptoms questionnaire

Burning post-sternal discomfort and pain	Yes	No
Non-burning chest discomfort and pain	Yes	No
Feeling of the food sticking, staying, and abnormal sensation of food passing through the esophagus	Yes	No
Difficulty and pain in swallowing	Yes	No
Lump sensation, foreign body sensation, burning sensation, pain in the throat	Yes	No
Snoring, nocturnal sleep apnea	Yes	No
Hoarse voice	Yes	No
Pain in the upper abdomen (before/after meals)	Yes	No
Bloating	Yes	No
Food nausea and nausea	Yes	No
Hiccups, belching	Yes	No
Sour/bitter taste in the mouth	Yes	No
Dental caries, tooth erosion	Yes	No
Bronchiectasis, chronic bronchitis	Yes	No
Chronic or long-lasting cough	Yes	No
Asthma	Yes	No
Chronic obstructive pulmonary disease	Yes	No

### 2.4 Research methodology

#### 2.4.1 Grouping method

Participants in the study completed the RDQ and a questionnaire survey on extraesophageal symptoms under the supervision of doctors. RDQ scores were calculated, and a diagnosis of GERD was established if the total score was equal to or higher than 12. All participants also underwent 24-h MII-pH monitoring. Based on the symptom scale and 24-h MII-pH monitoring results, we divided the participants into the proximal reflux group and the distal reflux group. There were 67 cases in total, consisting of 39 cases in the proximal reflux group and 28 cases in the distal reflux group.

#### 2.4.2 Preparation before the examination

(1) Pre-procedural preparation for patients: Patients were required to be fasting and avoid drinking water for at least 6–8 h to prevent vomiting and aspiration. Before the examination, patients were required to also stop taking medications that affect esophageal motility and change the gastric pH value, PPI drugs, and other acid-inhibiting drugs for 2 weeks, and asked to remain off motility drugs for more than 1 week prior to the procedure;
(2) After the electronic gastroscopy, high-resolution manometry was arranged following an interval of more than 3 days;
(3) We collected the detailed medical history, including symptoms (heartburn, acid reflux, dysphagia, chest pain, and so on) and duration, heart disease, severe lung disease, operation history, PPI usage, and history of use of gastrointestinal motility drugs, and so on;
(4) Participants were given detailed information before the examination to make them understand the process of investigation, its necessity and importance, allay their fears and stress to make them cooperate better and successfully complete the examination;
(5) All participants provided their informed consent and signed the necessary documentation for the 24 h MII-pH monitoring and examination.

#### 2.4.3 Instructions for participants

(1) 24 h MII-pH monitoring would record all types of gastroesophageal reflux;
(2) Fasting for 6–8 h before the examination was required;
(3) Withdrawal requirements before the examination: PPI drugs and other acid inhibitors were stopped for 2 weeks and prokinetic drugs were stopped for more than 1 week prior to the procedure;
(4) Participants were given normal diet during the 24 h MII-pH and asked to avoid acidic or alkaline food;
(5) Participants were required to fill in the inspection log carefully and avoid smoking and drinking; if otherwise, this was to be recorded in the log;
(6) During the 24 h MII-pH monitoring, the catheter should be in place and prevented from falling off;
(7) Participants were requested to protect the examination instrument from damage as it was a valuable and delicate tool;
(8) Participants were requested to contact the team if there was no digital display or if they noticed any abnormal condition during the monitoring period;
(9) Participants were asked to return to the examination room the next morning to remove the recorder and hand over the inspection log.

#### 2.4.4 24 h pH impedance combined monitoring procedure

A new No.5 battery, electrode catheter, calibration solution of pH 4 and pH 7.01, data recorder, and satchel were prepared for each participant. The new No. 5 battery for the data recorder was fitted and the data recorder was placed in the satchel. Once the electrode conduit connector was connected to the interface of the corresponding color, the data recorder displayed the calibration mode. After setting the date and time, the confirm key was pressed to start soaking the catheter. After soaking for 60 s, the catheter was wiped dry and put into the pH 4.0 buffer to start the calibration. About 3 min later, after the calibration of pH 4.0, the catheter was put into clean water and wiped dry. After that, a buffer solution of pH 7.01 was added to start the calibration; After 3 min of pH 7.01 calibration, the catheter was placed in clean water and wiped dry. With this, the pH calibration of the catheter was completed.

A lubricant (liquid paraffin) was applied along a 20 cm length at the front end of the electrode catheter. The patient was asked to sit and the catheter was inserted into the nasal cavity. When the catheter was inserted about 15 cm into the pharynx, the patient was instructed to breathe normally and swallow. The catheter was inserted into the esophagus in parallel to the patient swallowing, until it was 5 cm above the LES, and allowed to enter the stomach first and then pulled back. The position of LES was determined by the change in pH value of the instrument and compared with the position during manometry. After determining the position, the catheter was fixed, the strap position was adjusted, and the “start recording” button was pressed.

The doctor recorded the starting time and guided the patient on how to fill in the record log. After the 24-h monitoring, the patient returned the instrument. The data were imported into the data analysis software in the computer along with the patient information and saved. After confirming that the 24-h data were complete, the participants were requested to stay for half an hour and then leave. The data were transferred to the corresponding patient’s file, double-clicked to enter the curve interface, and the system automatically analyzed the data and gave the report.

#### 2.4.5 Observation index

Depending on the pH value of the reflux, it was divided into acid reflux (pH < 4), weak acid reflux (pH 4–7), and non-acid reflux (pH > 7). According to the impedance value of the reflux, reflux was divided into liquid reflux (at least two continuous impedance channels appeared retrogradely from the end impedance channel, and the impedance value decreased by more than 50%), gas reflux (at least two impedance channels appeared, and the impedance value increased by more than 500 Q), and mixed reflux (gas reflux occurred immediately before or simultaneously with liquid reflux). Esophageal acid exposure time (AET) refers to the percentage of episodes of pH < 4 in the total monitoring time as measured by the pH electrode 5 cm above LES. AET > 4.2% indicated pathological acid reflux.

In this study, the main acquisition indicators included reflux, total acid exposure time percentage, percentage of episodes of pH < 4 in the total time which is the most effective indicator to observe pathological acid reflux, acid exposure time, frequencies of gas-liquid mixed reflux, acid, weak acid, and non-acid reflux, liquid, gas, and mixed reflux, proximal reflux, and distal reflux, symptom correlation index, and DeMeester score, amongst others.

DeMeester score [17]: According to the longest number of reflux episodes (>5 min), the number of episodes of reflux, the number of episodes (percentage) of pH < 4 in supine position, the number of episodes (percentage) of pH < 4 in upright position, and the number of episodes (percentage) of total pH < 4. If the final comprehensive score was more than 14.72, it was diagnosed as pathological acid reflux. If the symptom correlation index was >50% and symptom correlation probability was >95%, it indicated that the symptoms were related to reflux, and reflux referred to all types of reflux events.

#### 2.4.6 Statistical analysis

Data analysis was performed using SPSS 23.0 statistical software. If the measurement data were normally distributed and showed homogeneity of variance, they were presented as mean value ± standard deviation. In this case, an independent sample *t*-test was used to compare the data between groups. If the measurement data did not follow a normal distribution, quartiles (lower quartile, median, and upper quartile) were used to represent the data. Non-parametric rank sum tests were employed to compare the data between groups in such cases. Count data were presented as the number of cases and percentages. A chi-square test was used to compare count data between groups, with a significance level of *P* < 0.05 considered statistically significant.


**Ethics approval and consent to participate:** This study was conducted with approval from the Ethics Committee of the First Affiliated Hospital of Henan University of Science and Technology. This study was conducted in accordance with the declaration of Helsinki. Written informed consent was obtained from all participants.

## 3 Results

### 3.1 Comparison of clinical baseline data

Based on the symptom rating table and 24-h MII-pH monitoring results, the 67 patients in our study were divided into two groups: the proximal reflux group and the distal reflux group. The proximal reflux group consisted of 28 patients (41.8%), including 17 males and 11 females, with ages ranging from 34 to 77 years and a mean age of (57.46 ± 10.83) years. The average body mass index (BMI) was (20.92 ± 3.21), and the RDQ score was 14 (13, 15.25).

In the distal reflux group, there were 39 patients (58.2%), comprising of 23 males and 16 females, with ages ranging from 18 to 77 years and a mean age of (52.31 ± 11.85) years. The average BMI was (21.02 ± 2.56), and the RDQ score was also 14 (13, 15.5). There were no significant differences in gender, age, and BMI between the two groups (Table 4). RDQ scores of both groups were ≥12, and the statistical analysis showed no significant difference between them (details are given in Table 5).

**Table 4:** General information of patients included

	Distal reflux group	Proximal reflux group	*P*
Number of cases (persons)	28	39	—
Male/female ratio (person)	17/11	23/16	0.886
Age	57.46 ± 10.83	52.31 ± 11.85	0.078
BMI (kg/m^2^)	20.92 ± 3.21	21.02 ± 2.56	0.800

**Table 5:** RDQ score of proximal and distal reflux patients

	Proximal reflux group	Distal reflux group	Statistics	*P*
RDQ	14.00 (13.00, 15.50)	14.00 (13.00, 15.25)	*z* = −1.466	0.143

### 3.2 Manifestation of symptoms

Based on the results of 24-h MII-pH monitoring, in addition to experiencing typical symptoms, the proximal reflux group frequently reported extraesophageal symptoms such as foreign body sensation in the throat and chronic cough. The number of episodes of weak acid reflux, gas-liquid mixed reflux, total number of proximal refluxes, number of proximal weak acid reflux, and number of proximal non-acid reflux in GERD patients with extraesophageal symptoms were higher than those in GERD patients without extraesophageal symptoms. There were no significant differences in the total reflux times of pH ≤ 4, the percentage of total reflux times of pH ≤ 4, the longest reflux time, DeMeester score, acid reflux episodes, liquid reflux episodes, proximal acid reflux episodes, or standing and lying position-related reflux episodes.

### 3.3 24 h pH impedance monitoring results

The frequency of acid reflux in the proximal reflux group was 37 times (31, 41), which was more than that in the distal reflux group of 35 (30, 39), and the difference was statistically significant (*z* = −3.456, *P* < 0.05). The number of gas reflux episodes in the proximal reflux group was 27 times (23, 31.5), which was more than 19 (16, 23) in the distal reflux group, and the difference was statistically significant (*z* = −22.023, *P* < 0.05). The number of mixed reflux episodes in the distal reflux group was 29 (24.75, 35.25), which was higher than 21 (18, 25) in the proximal reflux group (*z* = −24.444, *P* < 0.05). In addition, there was no significant difference in the number of times of weak acid reflux, non-acid reflux, and liquid reflux between the two groups (details are given in Tables 6 and 7).

**Table 6:** Comparison of acid and base reflux in proximal reflux group and distal reflux group

Group	Number of cases	Reflux acidity and alkalinity		
		Acid reflux	Weak acid reflux	Non-acid reflux
Distal reflux group	28	35 (30, 39)	31 (25.75, 35)	4.5 (2.75, 6.25)
Proximal reflux group	39	37 (31, 41)	31 (25.5, 36)	5 (3, 6.5)
Statistics	—	*z* = −3.456	*z* = −2.517	*z* = −1.725
*P*	—	*P* = 0.001	*P* = 0.120	*P* = 0.084

**Table 7:** Comparison of physical properties between proximal reflux group and distal reflux group

Group	Number of cases	Reflux acidity and alkalinity		
		Liquid reflux	Gas reflux	Mixed reflux
Distal reflux group	28	20 (17, 26.25)	19 (16, 23)	29 (24.75, 35.25)
Proximal reflux group	39	20 (18, 24.5)	31 (25.5, 36)	21 (18, 25)
Statistics	—	*z* = −2.312	z = −22.023	*z* = −24.444
*P*	—	*P* = 0.210	*P* = 0.000	*P* = 0.000

## 4 Discussion

In this study, we compared the pH of reflux between the proximal reflux group and the distal reflux group and found that the frequency of weak acid reflux in GERD patients with extraesophageal symptoms was significantly higher than that in the typical GERD group, which can explain why some patients only had cough symptoms but no obvious acid reflux, heartburn, and other typical manifestations. At the same time, compared with the typical GERD group, the number of episodes of gas-liquid mixed reflux in GERD patients with extraesophageal symptoms was more, and the total number of episodes of proximal reflux, the number of episodes of proximal weak acid reflux, and the number of episodes of proximal non-acid reflux were significantly higher, indicating that due to the lighter gas mass, it was easier for the gas-liquid mixed reflux to move to the proximal esophagus than the liquid reflux.

As the last barrier of anti-reflux, the high pressure of the upper esophageal sphincter (UES) can cause a foreign body sensation in the pharynx and even dysphagia. However, the low pressure of UESP makes it easier for the reflux to break through the anti-reflux barrier of the UES and enter the pharynx and larynx, which can cause cough symptoms by directly stimulating the cough receptors in the pharynx and larynx. Under normal circumstances, cough symptoms are a protective mechanism for the body, which can, to a certain extent, prevent reflux from being inhaled into the airway by mistake. Thus, it increases the risk of cough caused by direct stimulation of the airway by reflux inhalation, aspiration pneumonia, and even asphyxia.

Some research results have proved that [18] there is a relationship between the occurrence of esophagitis and gastroesophageal reflux. Acids in reflux can cause esophageal squamous epithelium damage and produce inflammatory reaction. At present, it has been confirmed in GERD patients and RE models that inflammatory products that cause esophagitis include prostaglandin and reactive oxygen species [19]. This study provides a reference for further research on esophagitis caused by gastroesophageal reflux by exploring the substances of proximal reflux and distal reflux.

Current research and understanding pertaining to GERD is that esophageal motility disorders are generally considered as due to the following factors: (1) Low or lack of LES pressure, transient LES relaxation; (2) The function of clearing esophageal reflux is weakened due to esophageal motility disorder; (3) Changes in esophagogastric angle (angle of His); (4) The barrier function of esophageal mucosa is damaged; (5) The changes in mucosal function in the cardia; (6) Psychological factors.

Among these, the dysfunction of esophageal anti-reflux barrier is one of the important factors in the pathogenesis of GERD, mainly manifested as transient relaxation of LES, decreased LES pressure, and ineffective esophageal movement. However, some studies suggest that the induction of cough is related to the retention time of reflux in the esophagus but not to the acidity of reflux. Gastroesophageal reflux cough, as a form of GERD, with cough as the main symptom, is generally considered as one of the most common causes of chronic cough, like cough variant asthma, upper airway cough syndrome, and eosinophilic bronchitis [20].

The typical symptoms of GERD are heartburn and acid reflux, and extraesophageal symptoms are also common in GERD patients. Extraesophageal symptoms refer to a series of symptoms caused by the reflux of gastric contents above the UES [21,22]. Extraesophageal symptoms of GERD may exist alone or simultaneously with typical symptoms [23]. In literature, GERD extraesophageal symptoms are referred to by using a variety of different labels such as laryngopharyngeal reflux, posterior laryngitis, reflux laryngitis, supraesophageal reflux, extraesophageal reflux, and gastroesophageal laryngeal reflux, amongst others [24].

Patients with GERD commonly experience a variety of extraesophageal symptoms, including throat clearing, chronic cough, voice hoarseness, excessive laryngeal mucus, postnasal drip sensation, dysphagia, chronic cough, burning sensation in the throat, sensation of a foreign body in the throat, and so on. The American Broncho-Esophagological Association found that the most relevant symptoms of reflux were clearing the throat (98.3%), intractable cough (96.6%), heartburn or dyspepsia (95.7%), change in quality of the voice (94.9%), sensation of a foreign body in the throat (94.9%), choking asphyxia (90.7%), and dysphagia (86.3%) [25]. Extraesophageal symptoms of GERD are also very common in children. In children, gastroesophageal pharyngeal reflux can lead to apnea, recurrent upper respiratory tract infections, laryngeal chondromalacia, subglottic stenosis, otitis, and other conditions [26].

Compared to the typical symptoms of GERD, extraesophageal symptoms tend to occur more frequently in the upright position during the daytime, while typical symptoms are more commonly experienced in a regular pattern during the supine position at night [27,28]. In a study conducted by Lewin et al. in 2003 [29], they found that among 34 patients with GERD and extraesophageal symptoms, reflux episodes occurring in the upright position accounted for 91% of cases, which aligns with the distinction between extraesophageal and typical GERD symptoms. With the global attention and continuous research on GERD, there has been steady research on GERD in China based on the actual situation of Chinese people. This has resulted in the establishment of the consensus for GERD treatment in China through the efforts of the dynamic group of experts from the gastrointestinal society of the Chinese Medical Association.

Currently, there are limited studies both domestically and internationally focusing on reflux types in patients with GERD. More large-scale multicenter clinical studies and related animal experiments are needed to explore the mechanisms and influencing factors of reflux, aiming to enhance the diagnosis and treatment efficacy of GERD.

## 5 Conclusion

The proximal reflux group exhibited a significantly higher frequency of acid reflux and gas reflux compared to the distal reflux group, indicating that acid reflux and gas reflux were more predominant in the proximal region. The number of episodes of mixed reflux in the distal reflux group was more than that in the proximal reflux group, and the difference was statistically significant, which confirmed that the distal reflux group was predominated by mixed reflux episodes. The number of episodes of weak acid reflux and gas-liquid mixed reflux in patients with GERD who had chronic cough were significantly more than those in the control group. This finding suggests that symptoms such as acid reflux and heartburn may not be apparent in some patients. The increased frequency of proximal weak acid and non-acid reflux further supports the possibility of cough triggered by throat stimulation.

## Abbreviations

AET: acid exposure time
GERD: gastroesophageal reflux disease
LES: lower esophageal sphincter
PPI: proton pump inhibitor
RDQ: questionnaires on reflux disease
RE: reflux esophagitis
UES: upper esophageal sphincter
24 h MII – pH: 24-h esophageal multichannel intraluminal impedance monitoring combined with pH-metry
